# Natural Cellulose Nanofibers As Sustainable Enhancers in Construction Cement

**DOI:** 10.1371/journal.pone.0168422

**Published:** 2016-12-22

**Authors:** Li Jiao, Ming Su, Liao Chen, Yuangang Wang, Hongli Zhu, Hongqi Dai

**Affiliations:** 1 Department of Pulp and Paper Engineering, Nanjing Forestry University, Nanjing, Jiangsu, China; 2 Department of Chemical Engineering, Northeastern University, Boston, United States of America; 3 Department of Mechanical and Industrial Engineering, Northeastern University, Boston, United States of America; 4 Department of Civil Engineering, Nanjing Forestry University, Nanjing, Jiangsu, China; East China Normal University, CHINA

## Abstract

Cement is one of the mostly used construction materials due to its high durability and low cost, but it suffers from brittle fracture and facile crack initiation. This article describes the use of naturally-derived renewable cellulose nanofibers (CNFs) to reinforce cement. The effects of CNFs on the mechanical properties, degree of hydration (DOH), and microstructure of cement pastes have been studied. It is found that an addition of 0.15% by weight of CNFs leads to a 15% and 20% increase in the flexural and compressive strengths of cement paste. The enhancement in mechanical strength is attributed to high DOH and dense microstructure of cement pastes after adding CNFs.

## Introduction

**Fig 1:**
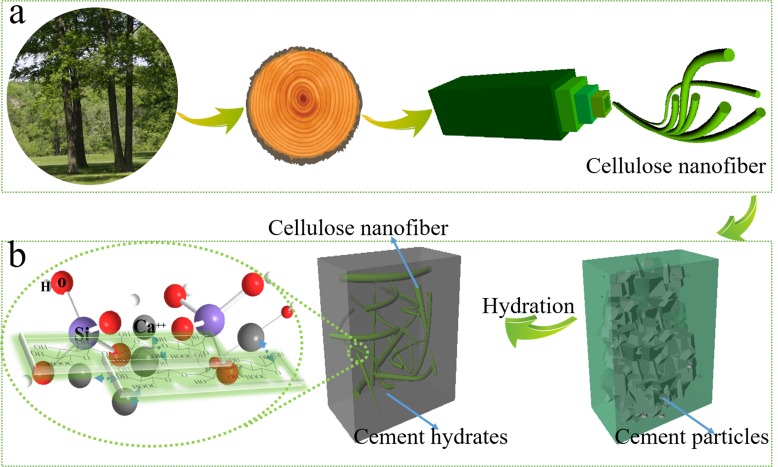
Schematic illustration of the hierarchical structure of a tree and the interaction between cellulose nanofibers and cement particles. (a) The hierarchical structure of celluloses. (b) A network of cellulose nanofibers and cement formed with carboxyl groups and cement hydrates.

Cement composite is one of the mostly used construction materials, but the use of cement based material in dams and long-span bridges is limited due to its brittleness [1–3]. Although a variety of fibers have been added into cement composites to improve their tensile strength, toughness, and energy absorption capacity [4–7], these fibers are limited for poor interface, low corrosion resistance, and high cost, etc. For instance, the bonding of glass fibers to cement is not strong enough, and glass fibers have low alkaline resistance [8,9], and cannot provide flexural, shear, and compressive forces [10,11]. Carbon and polymer fibers made from high energy consumption process are expensive [12]. Natural fibers have also been used to improve the mechanical properties of cement composites. The use of the naturally-derived fibers minimizes carbon footprint of infrastructural materials, -also provide excellent mechanical properties at low cost [13,14]. An addition of 2–16% (mass) millimeter long cellulosic fibers leads to 20–50% enhancement of flexural strength and fracture toughness of cement composites [15–19]. While, there are some issues related to natural fibers in cement composites. Fiber components such as lignin, hemicelluloses, pectin, and soluble sugars degrade in the alkali cement environment, leading to low durability [20–22]. Micrometer-sized natural fibers can easily aggregate, which creates weak bonding between fiber and cement hydrates, causing stress concentration at interface of fiber and cement [23]. It is reasonable to postulate that the mechanical properties of cement composites will be further improved if the selected natural fibers have high mechanical strength but not aggregate when mixed with cement particles. Cao reports that the flexural strength of cement paste increases with the addition of cellulose nanocrystals (CNCs). The degree of hydration of the cement paste increases that the mechanism proposed is steric stabilization and short circuit diffusion [24]. However, cellulose nanocrystals are typically prepared by acid hydrolysis, the most amorphous region of cellulose is removed and the yield of cellulose nanocrystals is only 20–30% [25–27]. Given that, cellulose nanofibers is an alternative, prepared by breaking down organized hierarchically natural cellulose fibers to nanofibers via mechanical and chemical process to break the hydrogen bonds at high yield, as shown in Fig 1(A).

Typically technology, cellulose nanofibers are prepared by 2, 2, 6, 6-tetramethylpiperidine-1-oxyl radical (TEMPO)-mediated oxidation system and mechanical homogenization, primary hydroxyl groups from the cellulose fibril are oxidized to carboxylate groups on the cellulose fibril surfaces. The introduction of carboxylate groups produce a negative charge on the surface of cellulose fibers, making the cellulose nanofibers suspension uniformly dispersed via the electrostatic repulsion force avoiding aggregation. Cellulose nanofibers not only have active chemical property endowed by the hydroxyl groups and carboxylate groups, but have excellent mechanical properties, for example, the elastic moduli of single microfibers prepared by TEMPO-oxidation is 145.2±31.3 GPa [28], which widely used to improve the mechanical property of polymers, such as chitosan, polylactic acid [29–31].

In this study, we added cellulose nanofibers to cement paste, and the mechanical strength of the cement was effectively enhanced by a low fraction of cellulose nanofibers. The porosity between cement hydrates and degree of hydration play a crucial role in the strength of cement, this work studied the effect of cellulose nanofibers on the porosity and degree of hydration of cement hydrates. The active hydroxyl groups and carboxyl groups from cellulose form hydrogen bonds or bridging compound with cement hydrates, the microstructure of cement pastes are effectively improved, as shown in Fig 1(B) and enhance the mechanical strength of composite.

## Results

### Effect of CNF fraction on the early hydration interaction of cement particles

**Fig 2:**
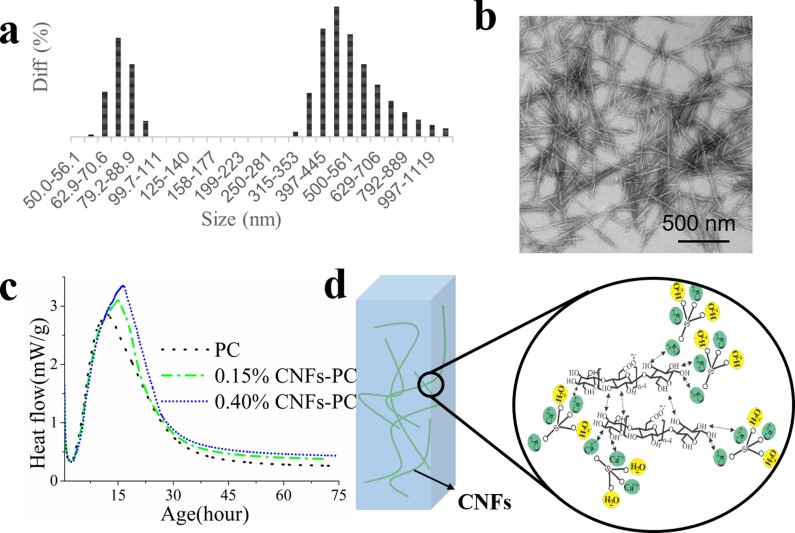
The morphology of cellulose nanofibers and the effect of CNFs on early hydration of cement particles. (a) Size distribution of CNFs; (b) TEM image of CNFs; (c) Heat flow curves of CNF-cement pastes for the first 72 hrs’ hydration; (d) Scheme of CNFs promoting the setting of the cement paste by coordination with Ca^2+^.

Cellulose nanofibers are isolated from wood using a combined TEMPO oxidation and homogenization approach [32–34]. The dimensions of CNFs range from 50–90 nm in diameter and 400–800 nm in length as in Fig 2(A) and 2(B). Isothermal calorimetry (IC) is used to examine the reaction between CNFs and cement by measuring heat flow rate. Fig 2(C) shows the heat flow curves of a reference (named as PC) and cement pastes containing 0.15 and 0.4% (mass) of CNFs in the first 72 hours of hydration aging. At 10 h there is no significant difference for samples with and without CNF addition. This is because that the exposed surface of cement particles dominates heat release, and there are sufficient water around particles for hydration [35]. At later ages, the addition of CNFs lengthens induction periods and delays peak heat flows of cement pastes. The peak heat flow is delayed up to 8 hrs with addition of 0.15% CNFs. At this time, both the exposed surface of cement particles and water fractions affect heat release rate [35,36]. Active groups on cellulose macromolecules such as hydroxyl and carboxyl groups are hydrophilic [27]. The oxygen atom in hydroxyl and carboxyl group has unpaired electrons, which can react with calcium ion and form a hydrophilic complex that can adsorb on cement particles as in Fig 2(D). Thus, the number of active sites between cement particles and water decreases, and the distances between cement particles increase [37]. The rates of formation of calcium silicate hydrate (CSH) and calcium hydroxide (CH) slow down [16,38–41]. The adsorption and complexation effects reduce the surface activity of the hydration products, and retard production of calcium hydroxide crystals, The cement particles with CNFs took longer time to hydrate with the increase of CNFs fraction [37]. For ongoing hydration, adding CNFs in dispersion of cement particles leads to more hydration, and more heat release [35].

The effect of CNFs addition on early hydration of cement is examined by measuring water consistency and setting time using the Vicat test. Table 1 shows the results of the Vicat test. Water consistency increased with addition of CNFs, which is attributed to adsorption by hydroxyl groups in CNFs, CNFs have high water retention value [42]. The setting times of cement pastes are strongly affected by water/cement ratio, which is affected by added CNFs [37]. The initial and final setting times increase by ~100 and 90 mins, respectively, with the addition of 0.40% of CNFs.

**Table 1:** Water consistency and initial and final setting times for cement pastes.

CNFs fraction (%)	Water consistency(%)/ Error Bar (%)	Setting time (min)/Error Bar (%)	
		Initial	Final
0	29.78/ 1.49	170/ 2.22	219/1.95
0.05	30.22/1.51	178/ 2.34	228/2.01
0.10	30.89/1.55	190/3.01	236/3.05
0.15	31.33/1.63	222/2.56	273/1.67
0.25	32.67/1.75	250/2.77	288/2.47
0.40	34.44/1.46	272/1.86	310/3.50

### Mechanical properties of cement pastes

**Fig 3:**
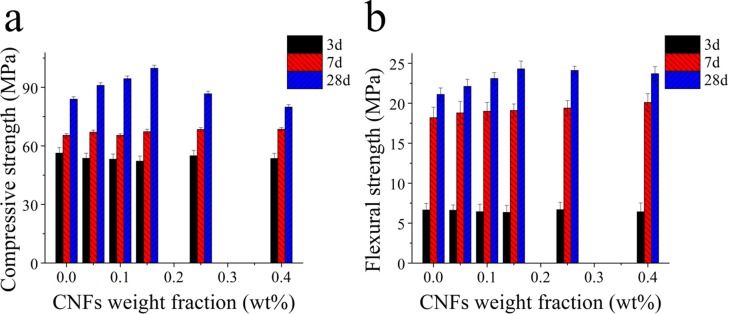
The flexural (a) and compressive (b) strengths of cement pastes with different CNF fractions addition at aging times of 3, 7, and 28 days.

Fig 3(A) and 3(B) shows the flexural and compressive strengths of cement pastes containing different fractions of CNFs measured after aging for 3, 7 and 28 days, respectively. After 3 days, cement pastes containing CNFs show no difference in flexural and compressive strengths. At early stages, the hydroxyl and carboxyl groups in the cellulose molecules react with Ca^2+^ to form complexes, which can delay induction period of hydration and also retard setting. With prolonged aging times of 28 days, the flexural and compressive strengths of cement pasts with 0.15% CNF increased 15% and 20%, respectively. This is because CNFs are a class of hydrogels with a high water absorption. After longer hydration aging, CNFs release water into its surrounding region, and contribute to hydration of un-hydrated cement particles, which can improve the microstructure and mechanical properties of cement pastes, in which the role of CNFs is similar to superabsorbent polymers [43,44]. The mechanical strength of cement pastes decrease at high CNF fractions. As their fractions increases, CNFs tend to form clumps, forming weak bonding interfaces that can promote stress concentration. The weak interfaces are detrimental to the mechanical strength of the cement pastes [45,46].

The degree of hydration and microstructure are critical components in determining the mechanical properties of cement pastes [47,48]. We measured the effect of CNFs addition on degree of hydration and microstructure in order to understand the reasons behind the observed improvements in flexural and compressive strength.

### Degree of hydration

**Fig 4:**
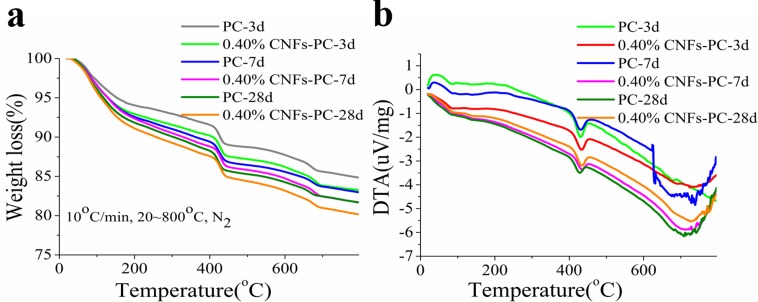
DTA-TGA results of cement pastes with different CNF weight fractions from 100 to 800°C.

One key behavior strongly related to the performance of cement pastes is hydration. The hydration reaction of cement pastes represents the mass conversion of different phases and correlates strongly with microstructure formation. The microstructure then determines the physical properties of cement pastes. The chemical bound water (CBW) from the harden cement pastes was investigated to measure the degree of hydration (DOH) by differential thermal analysis (DTA) combined with thermogravimetric analysis (TGA). In hydration process, cement paste will fully react and bind with water, forming CBW. For the cement pastes, higher CBW, higher DOH and more cement hydrates formed. In this study, CBW was measured by the lost mass between 100 to 800°C by igniting sample. Two important parameters needed to quantify the degree of hydration are the amount of chemically bounded water (W_b_) and the weight loss corresponding to decomposition of calcium hydroxide (CH_loss_) [49]. Fig 4(A) presents weight loss between 100 and 800°C, normalized to the mass at 100°C. Table 2 indicates that the weight loss increases with increasing fraction of CNFs. The weight loss of the reference cement paste is 19.3%, compared to a 21.0% loss from the cement paste containing 0.40% CNFs. The decrease in mass between 400 and 500°C observed in Fig 4(B) is attributed to decomposition of Ca(OH)_2_. The weight loss difference between two samples is 1.7%, indicating that more hydration products are produced with addition of CNFs.

**Table 2:** W_CH_ and W_b_ contained per gram cement paste with different CNF weight fractions.

Weight (g)	3d		7d		28d	
	ref	0.40%	ref	0.40%	Ref	0.40%
w _CH_	2.7%	3.0%	2.8%	3.2%	2.9%	3.3%
w _b_	16.3%	17.5%	18.1%	19.2%	19.3%	21.0%

W_CH_: Weight of CH per g cement paste; W _b:_ Weight of CBW per g cement paste.

CBW can be calculated by dividing the weight loss in the range of 100 to 800°C by the final weight of the material. With the assumption that the CBW is 0.23g per unit gram of the cement when fully hydrated, DOH can be easily obtained by normalizing the mass of CBW per unit gram of un-hydrated cement with 0.23g [49].

Table 3 shows DOH of cement pastes with different CNF fractions at different aging times. The results indicate that the DOH of cement pastes improves regardless of aging time. An explanation for the improvement of DOH with CNF additions at the same w/c ratio is that CNFs allow for a more efficient reaction between cement particles and water. CNF improves uniformity of cement particle-water mixture, reducing the amount of un-hydrated particles.

**Table 3:** Degree of hydration of cement pastes at different aging times.

Age	3d		7d		28d	
	Ref	0.40%	Ref	0.40%	Ref	0.40%
α_t_(%)	70.9	76.1	78.7	83.5	83.9	91.3

### Microstructure of cement pastes

**Fig 5:**
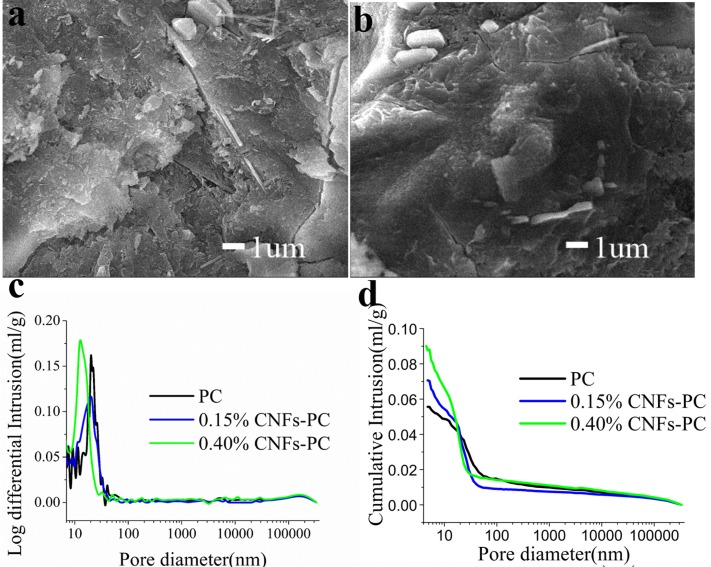
The microstructure of cement pastes after aging 28 days. (a) SEM image of the cement paste. (b) SEM image of cement paste with 0.15% of CNFs; (c) and (d) Pore diameter distributions of cement pastes with different CNF content.

Scanning electron microscopy is used to characterize the microstructure of cement pastes at different fractions of CNFs. Fig 5 shows the microstructures of the reference and cement paste with 0.15% of CNFs. More microcracks can be found in the reference (Fig 5(A)) than in the cement pastes with 0.15% CNFs (Fig 5(B)). A mercury intrusion porosimeter (MIP) is used to quantitatively compare the porosity of cement pastes containing CNFs. The pore size distribution and cumulative volume of cement pastes containing different fractions of CNFs measured by MIP are shown in Fig 5(C) and 5(D). Both the pore size and pore size distribution are affected by addition of CNFs. The pore size decreases with increasing CNF content, as shown in Table 4. The pore size for the cement paste with 0.40% CNFs is in the range from 10 to 22nm, compared to 15 to 40 nm for the reference. The mean pore size decreases with addition of CNFs, reaching minimum at 0.15% CNFs. The porosities for the reference, 0.15% CNF-paste, and 0.40% CNF-paste are 13.9%, 13.5% and 16.2%, respectively, suggesting that the total amount of pores decreases with CNF addition. The swelling CNFs with a high water adsorption releases water to promote hydration of un-hydrated cement particles, which can form more cement hydrates and reduce the macro-pores or micro-pores, so there are smaller pores and the porosity of cement pastes is reduced with modest addition of CNFs.

**Table 4:** The effect of CNFs addition on the pore structure.

CNFs fraction (%)	Mean pore size(nm)	Porosity(%)	Critical pore diameters(nm)
0	14.0	13.9	21.9
0.15	13.1	13.5	21.1
0.40	13.5	16.2	12.3

## Discussion

Many researchers have investigated the variety of fibers on the improvement of cement composites. Natural renewable cellulose fibers get a lot of attention due to their environmental and low cost characters. However, millimeter and micrometer-sized cellulose fibers have aggregation issue which is bad for the mechanical property of cement composites. Therefore, we pay our attention to the cellulose nanofibers. In addition, in comparison to the cellulose nanocrystals as reinforce for cement pastes, the cellulose nanofibers have the high yield and low cost, which is economic for civil engineering at large consumption.

**Fig 6:**
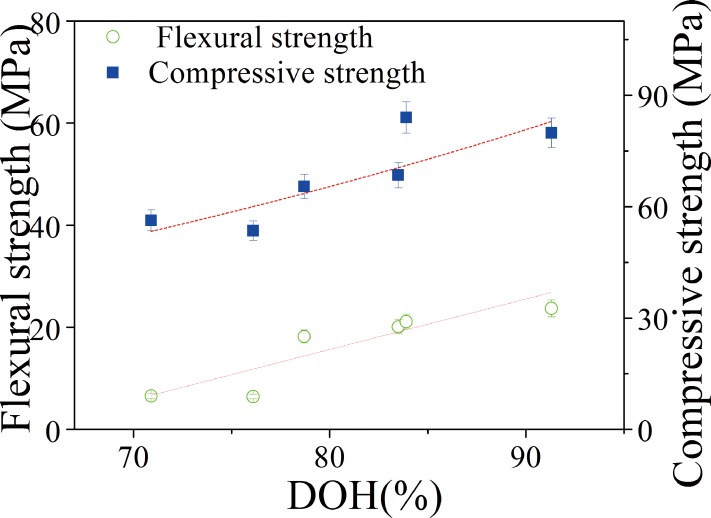
The relationship between mechanical strength (flexural strength and compressive strength) and DOH.

The mechanical strengths of cement pastes are improved with modest addition of cellulose nanofibers. The strengthening is proposed to the high level of DOH and reduced porosity. This is clear when the flexural and compressive strengths are plotted against DOHs obtained from DTA-TGA. Fig 6 shows the relationships between the mechanical strength and DOH. Although there are some exceptions, the mechanical strength largely increases with DOH. This can be explained by the fact that mechanical strengths are also affected by other factors than the DOH. Though cement pastes have higher DOH at high CNF fractions, but porosity also increase, both are detrimental to the mechanical strength [43]. So, multiple factors should be taken into account to improve overall mechanical strengths.

To understand the mechanism for the improvement in the mechanical strength of cement pastes, The following reasons are speculated. First, the DOH of cement pastes increased with the addition of CNF fraction. Higher DOH, more hydrates formed, which promotes the improvement of mechanical strength [50]. Second, CNFs have high water retention property which prolongs the setting time of cement pastes, and promotes more un-hydrated cement particles hydration, the microstructure of cement pastes is improved and less porosity form. As a result, a higher DOH and smaller porosity enhance the mechanical properties of the cement paste. [24]. However, there are some limitation, much more cellulose nanofibers easily cause the aggregation and decrease the workability of cement pastes, that is, the viscosity of fresh cement pastes increases which is consist with Catalina’s results, who has reported that cellulose microcrystalline particles decrease the workability of fresh cement paste and the hydration reaction was delayed [51]. Cellulose nanofibers can be used along with superplasticizer to relief the above issues. Therefore, there are still many works to do for the cellulose nanofibers as a reinforcement agent for cement composites.

## Materials and Methods

The cellulose nanofibers are prepared by using the combination of TEMPO–mediated oxidation system and mechanical homogenization [32,52]. The content of carboxyl groups on the surface of cellulose fibers was characterization by conductivity titration [53].

CNF-cement pastes were prepared by mixing CNF suspensions, water, and cement particles at different ratios. Experimental tests mainly evaluated how CNFs affected the properties of cement pastes, the mechanical performance of cement pastes, and the evolution of microstructure of cement pastes. Both the amount of water needed for standard consistency of cement pastes and the setting time via the Vicat test were characterized to evaluate the interaction and affinity of CNFs with cement particles. The DOH and microstructure of the cement played the most important roles in the mechanical performance of cement composites.

### Morphology of cellulose nanofibers

Meanwhile, CNFs were observed by JEOL JEM-1010 Transmission electron microscopy at 80 KV accelerating voltage. CNF suspension was deposited on carbon-coated TEM grids (300 mesh copper), and negatively stained with 1.5% phosphotungstic acid.

### Cement past elaboration

Ordinary Portland cement (PII 42.5 R) used in this study was provided by Nanjing Jiangnan Co., Ltd. The compositions and physical properties of Portland cement particles are shown in the Additional information (Table A in S1 File). Cement pastes were prepared at a constant water-to-cement ratio (w/c) of 0.35 with five different CNF fractions (0, 0.10%, 0.15%, 0.25%, and 0.40%). The CNF fraction was calculated based on CNFs weight with respect to the weight of the cement particles. Distilled water was first added to the CNF suspension, and mixing was induced by agitation for 15 min using a magnetic stirrer and then sonicated 15 min at 20% maximum power. Then, the cement particles and CNFs suspension were added to a blender and mixed at a speed of 500 rpm for 120 s. Next, the mixing was stopped for 15 s to scrape the wall and bottom of the bowl, followed by another 120 s of mixing at 600 rpm. Then, the fresh cement pastes were cast into 40×40×160 mm^3^ plastic rectangular molds and vibrated mechanically 120 times to decrease honeycombs and holes. The cement samples were sealed at 20±1°C and 95% relative humidity for curing. After aging for 24±1 hours, the rectangular samples were demolded and placed back in the above environment.

### Vicat test

A Vicat apparatus was used to determine the water quantity needed for a standard consistency of cement pastes containing different fractions of CNFs. The initial and final setting times of cement pastes with or without CNFs were also measured.

### Isothermal calorimetry

To test the effect of CNFs on the initial hydration reaction of cement pastes, the heat flow rate of cement pastes was measured using an 8-channel isothermal calorimeter (TAM Air from Thermometric AB, Sweden). After mixing the cement, different fractions of CNFs (relative to the cement weight) were added at a 0.5 water-cement ratio. The specimens were immediately transferred to plastic ampoules; the ampoules were sealed and placed into the chamber (maintained at 20±0.1°C) for testing. Before the start of data collection, isothermal conditions were held for 45 min to ensure equilibration, and the subsequent steady heat measurement was performed for 72 hrs.

### Mechanical strength testing

40×40×160 mm^3^ bars of cement paste was made as follows. The samples are unmolded after 24 hours, and preserved in water in a 20°C, 95% humidity chamber. Three-point bending tests were conducted in a microcomputer controlled machine by applying a 150 kN flexural load cell at a loading rate of 50 N/s. Compression tests were also carried out by imposing a loading rate of 2400 N/s on cement paste specimens using a microcomputer controlled machine, shown in Figure A in S1 File.

### Thermogravimetric analysis

To analyze the effect of CNF addition on the degree of hydration of cement pastes, cement pastes with 0%, 0.15%, and 0.40% (mass) of CNFs at aging times of 3, 7, and 28 days were subjected to thermogravimetric analysis (CH2 DTG-60AH). Before testing, the cement pastes were soaked in ethanol for 48 h to stop hydration and dried at 60°C in a vacuum oven for 48 hrs. Then, the samples were ground into powders, and 7.0 mg of powder was transferred into the DTA-TGA chamber for the measurements. All specimens were decomposed at a temperature range between 20 and 800°C at a heating rate of 10°C/min under nitrogen.

### Microstructural analysis

The microstructure of the cement pastes was investigated using field emission scanning electron microscopy (SEM, JSM-7600F, Japan). SEM was carried out on the fractured surface of the cement paste after mechanical testing. Prior to the measurement, samples were soaked in anhydrous ethanol for 48 h and then dried at 60°C in a vacuum oven for 48 hrs. The specimens were mounted on aluminum stubs with conductive carbon tape and sputtered with gold under vacuum at 20 mA for 2 min.

The porosity of cement paste composites was examined as follows. The pore volume and pore size analysis were performed on a mercury intrusion porosimeter (Micromeritics AutoPore 9500, America). The specimens were intruded with mercury and the quantity and pressure required for the intrusion were used to calculate the sizes and amount of pores within the sample according to the Washburn equation:

$$r=-2\frac{rcos\theta }{p}$$ (1)

where r is the pore radius (μm), γ is the surface tension of mercury (480 mN/m), θ is the contact angle between mercury and probe wall (140° as recommended by the manufacturer of the porosimeter), and P is the applied pressure (MPa) [54]. Three repeated measurements were conducted for each test.

## Supporting Information

S1 File Compositions of cement (Table A). The images for testing the flexural strength and compressive strength (Figure A).
(DOCX)
