# Supplementary material for: Natural Cellulose Nanofibers As Sustainable Enhancers in Construction Cement
Source: PLoS One. 2016 Dec 22;11(12):e0168422. doi: 10.1371/journal.pone.0168422 (PMC5179083; doi:10.1371/journal.pone.0168422)
Supplement: S1 File — The images for testing the flexural strength and compressive strength (Figure A). (DOCX) [file pone.0168422.s001.docx]

**Natural Cellulose Nanofibers as Sustainable Enhancers in Construction Cement**

**Supporting Information**

**Table A. Compositions of cement**

| CaO | MgO | Fe_2_O_3_ | Al_2_O_3_ | SiO_2_ | K_2_O | Na_2_O | SO_3_ | P_2_O_5_ | MnO | TiO_2_ | LOI | Blaine fineness |
| --- | --- | --- | --- | --- | --- | --- | --- | --- | --- | --- | --- | --- |
| 54.6305 | 3.2725 | 2.9167 | 6.6034 | 24.061 | 0.5617 | 0.1205 | 2.7257 | 0.0795 | 0.8466 | 0.4244 | 3.05 | 365 |


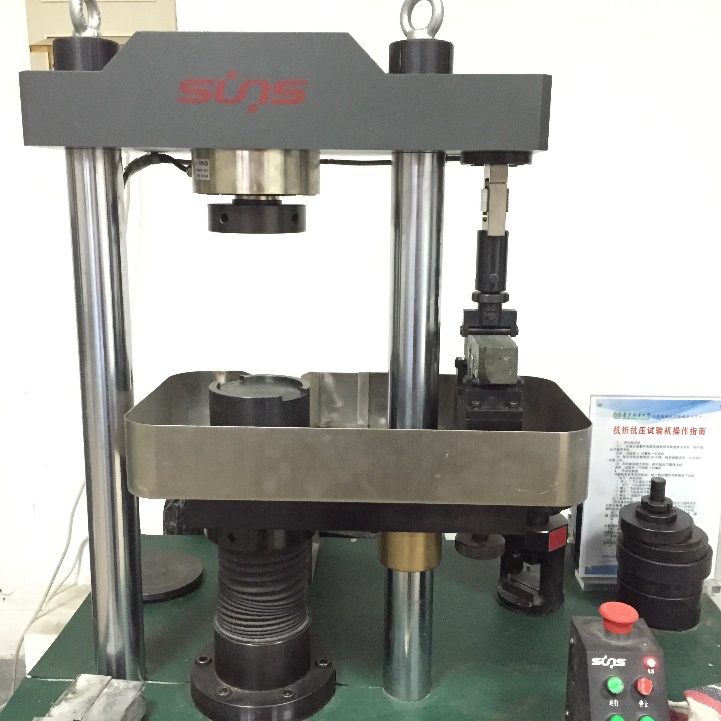


**Fig A. The images for testing the flexural strength and compressive strength**
